# TelePriming sentence production in aphasia

**DOI:** 10.3389/fnhum.2023.1274620

**Published:** 2023-11-09

**Authors:** Jiyeon Lee, Austin D. Keen, Ellis Farr, Sharon Christ

**Affiliations:** ^1^Aphasia Research Laboratory, Department of Speech, Language, and Hearing Sciences, Purdue University, West Lafayette, IN, United States; ^2^Department of Human Development and Family Science, Purdue University, West Lafayette, IN, United States

**Keywords:** aphasia, structural priming, sentence production, telerehabilitation, implicit learning

## Abstract

**Background:**

The application of videoconferencing to the assessment and treatment of aphasia has been rapidly increasing; however, there is a need to develop treatments targeting sentence production in persons with aphasia (PWA) that can be delivered through videoconferencing. Structural priming has received recent attention as a potential training method for PWA. We investigated the feasibility and efficacy of a collaborative structural priming task delivered *via* the internet, *TelePriming*, in facilitating sentence production in PWA and healthy adults.

**Method:**

In Study 1, young adults (YA), older adults (OA), and PWA participated in a collaborative dialogue-like priming task through videoconferencing, taking turns with an interlocutor (experimenter) to describe transitive action pictures with the goal of finding matching pictures. We measured whether participants produced more passive sentences to describe their picture after hearing their interlocutor produce passive compared to active sentences (primes). In Study 2, we compared the data from the OA and PWA of Study 1 (TelePriming) to different groups of OA and PWA, who completed the same priming task in person.

**Results:**

All three groups showed robust priming effects in Study 1, producing more passive sentences to describe target pictures after hearing the experimenter produce passive versus active sentences. In Study 2, when controlling for demographic information (age, education) and aphasia severity, TelePriming resulted in larger priming effects for OA and PWA, compared to the in-person priming task. Survey results revealed that both OA and PWA experienced increased comfort and satisfaction with using technology following the task.

**Conclusion:**

Interactive message-structure alignment processes remain largely intact in PWA, and the positive effects of structural priming in a collaborative communicative task are not diminished by remote delivery. The findings demonstrate the feasibility and validity of TelePriming in OA and PWA, laying experimental groundwork for future use of TelePriming in the assessment and treatment of clinical populations with limited access to face-to-face sessions.

## 1. Introduction

Aphasia is an acquired language processing disorder that is most often caused by stroke. In many persons with aphasia (PWA), the ability to map messages to language forms is impaired, resulting in varying degrees of deficits across the spectrum of communication from single-word to sentence-level utterances. Successful aphasia rehabilitation, therefore, requires personalized long-term care where PWA receive targeted treatments that address different goals as they move through the recovery process. Research in aphasia rehabilitation has made substantial advancements over recent decades, demonstrating the positive effects of speech therapy as a standard of care for aphasia even at chronic stages of recovery. However, compared to the numerous aphasia treatments available for improving naming abilities, very few treatments are available to treat sentence-level deficits (e.g., Poirier et al., 2021 for review; see also Brady et al., 2016). The few existing sentence treatments typically aim to improve patients’ ability to produce and comprehend sentences through explicit drill-based training of grammatical rules and hands-on manipulation of stimuli. For example, patients might be asked to reorganize cards with constituent phrases to construct a complete grammatical sentence following a clinician’s modeling and feedback (Rochon et al., 2005; Thompson and Shapiro, 2005). Such explicit metacognitive principles of learning, however, are not always effective for PWA, yielding only small treatment gains in some patients. Therefore, there is a need to expand the available treatment options that target sentence production deficits in aphasia.

Another critical need involves improving the accessibility of aphasia services. Many PWA have limited access to therapy or research opportunities due to various barriers, including physical immobility following stroke, geographic distance, and a shortage of clinicians or insurance coverage. Following COVID-19, telepractice—the remote delivery of assessment and treatment services provided using information technology and telecommunication systems—has been used ever increasingly to fill this gap in both research and clinical settings. As such, a growing number of studies have demonstrated the feasibility and validity of applying videoconferencing to aphasia assessments and treatments (Hill et al., 2009; Hall et al., 2013; Coleman et al., 2015; Øra et al., 2020; Jacobs and Ellis, 2021). Telepractice offers several benefits, including improved accessibility of cost-effective speech services for PWA and expanding research to include more diverse and underrepresented PWA (e.g., Øra et al., 2020; Jacobs and Ellis, 2021). Administration of standardized aphasia tests using videoconferencing, has shown comparable results to in-person testing, although the ability to assess certain skills (e.g., naming and paraphasias) may be affected by the mode of task delivery in severely impaired PWA (Hill et al., 2009; Dekhtyar et al., 2020). Studies have shown that computerized therapy programs or treatments delivered *via* videoconferencing provide an important cost-effective alternative to in-person therapy which is necessary for long-term care in PWA (Palmer et al., 2020; Braley et al., 2021; Jacobs and Ellis, 2021). Telerehabilitation of aphasia has exhibited a high level of feasibility and acceptability, as measured by protocol adherence, technical fault rate, and satisfaction of patients and clinicians (Øra et al., 2020).

However, current research on remote delivery of aphasia treatments is largely limited to treatments targeting naming ability or production of situation-specific functional scripts (Goldberg et al., 2012; Furnas and Edmonds, 2014; Meyer et al., 2016; Dial et al., 2019). These studies generally show significant gains on trained target stimuli (e.g., Dechêne et al., 2011; Fridler et al., 2012; Agostini et al., 2014; Furnas and Edmonds, 2014; Meyer et al., 2016; Woolf et al., 2016) and some generalization to untrained stimuli and tasks (Furnas and Edmonds, 2014; Dial et al., 2019). However, researchers have not yet examined the efficacy of remotely delivered sentence production treatments such as the TUF (Thompson et al., 2003) or Mapping Therapy (Rochon et al., 2005). This paucity of evidence could be due to methodological limitations as existing sentence production treatments rely heavily on complex hands-on manipulations of physical stimuli combined with multiple steps of instructions, which are difficult to deliver over the internet. Therefore, there is a need to develop treatments specifically targeting sentence production in PWA that can be easily delivered through videoconferencing. Structural priming has received recent attention as a potential training method to facilitate sentence production in PWA, and the priming paradigms do not require complex manipulation of stimuli. Therefore, the current study examines the efficacy of a collaborative structural priming task on the production of passive sentences in young adults (YA), older adults (OA), and persons with aphasia (PWA) when it is delivered *via* videoconferencing (i.e., TelePriming hereafter).

Structural priming is the tendency for language users to reuse a previously encountered (primed) sentence structure to facilitate their future sentence production or comprehension (Bock, 1986; Pickering and Ferreira, 2008). For example, if a speaker hears a passive sentence (prime), they are more likely to produce a passive sentence to describe a new transitive event (target), compared to when they hear an active prime. Structural priming is observed in numerous syntactic structures and languages, in both spoken and written modalities of communication, and in young children and older adults (Pickering and Ferreira, 2008 for review; Mahowald et al., 2016; Lee et al., 2022). Theories of structural priming suggest that through the process of priming, speakers learn to map a message onto syntactic forms based on prior linguistic experience, creating enduring experience-based adaptations to the production system across the life span (Bock and Griffin, 2000; Chang et al., 2006, 2012; Ferreira and Pickering, 2008). In support of the learning view of structural priming, studies have reported priming effects that persist even when multiple unrelated utterances intervened between the prime and target (Bock and Griffin, 2000; Bock et al., 2007). Priming effects have also been shown to cumulatively increase the production of primed structures over multiple trials or sessions (Kaschak, 2007; Kaschak et al., 2011, 2014; Heyselaar et al., 2021; van Boxtel et al., 2023), and in some cases, greater priming effects are observed in less frequent structures or less efficient speakers (Hartsuiker and Westenberg, 2000; Scheepers, 2003; Kaschak et al., 2011).

Interestingly, priming effects become particularly robust when the task involves two speakers collaboratively using language under a shared goal, compared to a single-speaker priming task where participants read or repeat a prime sentence and then describe a target picture. A commonly used collaborative priming paradigm is a scripted dialogue task wherein the participant takes turns describing pictures with a confederate conversational partner (who is often an experimenter) with a shared goal such as finding identical pictures (Branigan et al., 2000, 2007; Gruberg et al., 2019; Schoot et al., 2019). Unbeknownst to the participant, the partner describes their picture using a pre-determined (scripted) sentence structure such as a passive sentence, which serves as a prime. If priming is effective, the participant should use the same sentence structure that they heard their partner produce when it is their turn to describe a picture. This interactive syntactic alignment is hypothesized to facilitate information processing between interlocutors by creating shared “routines” or situation models in dialogue (Pickering and Garrod, 2004, 2007). Due to its social-functional role, structural priming effects are expected to be greater in dialogue-like tasks, rather than in a monologue or spontaneous speech (Branigan et al., 2000; Reitter and Moore, 2014; Schoot et al., 2019). In addition, priming in dialogue-like tasks is more likely to spread across different levels of linguistic representations (e.g., syntactic, lexical), predicting additive priming effects when lexical information is shared between the prime and target sentences. For example, priming effects are expected to be larger when the same verb is repeated between prime and target compared to when different verbs are used, i.e., the lexical boost effect (Hartsuiker et al., 2008; Menenti et al., 2012; Branigan and McLean, 2016).

Most interestingly, structural priming has been applied to clinical populations with language disorders, including both children and adults, demonstrating its potential as an intervention. Studies show that children with development language disorders produce complex utterances and grammatical forms more easily following priming experiences (Leonard et al., 2000; Miller and Deevy, 2006; Garraffa et al., 2015; see also Garraffa and Smith, 2022 for review). Children with autism show preserved ability to align linguistic expressions with their conversational partner in a dialogue-task (Allen et al., 2011). Relevant to the current study, rapidly increasing evidence suggests that structural priming creates not only immediate but also lasting and generalizable improvement in PWA. After hearing or repeating prime sentences, PWA are able to produce sentences that they could not produce otherwise more frequently (Saffran and Martin, 1997; Hartsuiker and Kolk, 1998; Yan et al., 2018), and these priming effects persist over intervening filler utterances (Cho-Reyes et al., 2016; Lee et al., 2019; Man et al., 2019). Priming experience in the production modality shapes the ways in which PWA interpret subsequent syntactically ambiguous sentences, suggesting that structural priming creates enduring changes in the central syntactic system (Keen and Lee, 2022). In a recent eyetracking structural priming study, van Boxtel and colleagues found that a group of PWA showed cumulative improvement in the production accuracy of passive sentences and the use of an advanced sentence planning strategy, even when they failed to show significant immediate priming effects (van Boxtel et al., 2023). Lastly, following structural priming training, PWA have shown significantly improved production of sentences that was maintained even after a week or a month, and there was some generalization to untrained stimuli and contexts (Lee and Man, 2017; Lee et al., 2023).

Most of the existing studies addressing priming in aphasia have used single-speaker monologue-based tasks. Only two studies have so far examined the effectiveness of collaborative priming in PWA (Lee et al., 2019; Man et al., 2019). In Lee et al. (2019), although a priming effect was found for young adults, PWA and OA failed to show significant syntactic alignment when they simply heard their confederate partner’s sentences; however, PWA and OA showed significant syntactic alignment when they also orally repeated the sentences that their confederate partner produced. The lack of a significant priming effect in the listening-only condition could be because Lee et al. (2019) included a longer lag (10–12 intervening fillers) between the prime and target and multiple syntactic structures (transitive, dative, and locative) within a single session. Man et al. (2019), on the other hand, found that PWA and OA showed significant priming in a scripted dialogue task in both immediate (0-lag) and lasting (2-lag) priming conditions, although the effects were reduced in PWA. In addition, OA showed a significant lexical boost effect when the verbs were repeated between prime and target sentences, but PWA failed to show such a lexical boost on priming.

Therefore, it is not entirely clear whether PWA show robust priming effects in the context of collaborative language tasks and how various factors such as the modality of task delivery (tele- vs. face-to-face interaction) or stimuli type (verb overlap vs. no overlap) might influence outcomes. Considering that social attention and communicative intent are known to affect robust priming found in collaborative tasks (Branigan et al., 2007; Schoot et al., 2019), it is important to test if TelePriming would be as effective as in-person priming in facilitating sentence production for PWA. If so, TelePriming would hold longer-term potential to be used as a novel intervention that can be easily delivered over the internet to treat sentence production deficits. From an implementation perspective, structural priming paradigms do not require involved hands-on manipulation of stimuli or complex metacognitive instructions and steps and thus might be more suitable to be delivered over videoconferencing compared to existing sentence treatment approaches. Additionally, given the premise that priming spreads across different linguistic representations in dialogue (Pickering and Garrod, 2004), it is also important to examine if verb overlap boosts priming effects in PWA during TelePriming. If positive lexical boost is found, the findings would inform what types of stimuli might lead to greater priming-induced improvement in PWA, when structural priming in a dialogue context is used as an intervention.

Two studies were conducted to examine the feasibility of the TelePriming paradigm in facilitating sentence production of PWA. Study 1 investigated if young adults (YA), older adults (OA), and PWA would show increased production of passive sentences in a dialogue-like priming task, when it is delivered over videoconferencing. In addition, we used brief surveys to gauge participants’ experience with the online research format to see whether TelePriming was, besides being effective, also enjoyable. Given the burgeoning evidence that supports the feasibility and validity of telepractice in the assessment and some treatments of aphasia along with previous structural priming studies that demonstrate the success of internet-based tasks in monologue priming with healthy adults (Smith and Wheeldon, 2001; Corley and Scheepers, 2002; Bahadir and Polinsky, 2012; Getty and Fraundorf, 2023), we predicted that all three groups would show significant TelePriming effects in Study 1, although the priming effects might be smaller in PWA. We also predicted that OA and PWA would report increased positive experiences with the technology after the completion of the study, although they might be less ‘comfortable’ at the beginning of the study compared to the YA group. In Study 2, we compared the data from the OA and PWA of Study 1 (TelePriming) to different groups of OA and PWA, who completed the same priming task in person in a laboratory setting. We predicted that the participants would show comparable priming effects between the two task modalities when demographic information (age, education) and aphasia severity were controlled for in the between-subject design. Lastly, to test whether participants showed lexical boost effects in both studies, the same verb was repeated between the prime and target for half of the experimental trials, while the other half of the prime-target pairs contained different verbs.

## 2. Study 1

### 2.1. Participants

Fourteen healthy young adults (YA), 14 healthy older adults (OA), and 10 persons with aphasia (PWA) were recruited for Study 1. Data from 2 YA and 2 OA were excluded as 3 of them (1 YA, 2 OA) identified the purpose of the task during the study debriefing and showed 100% priming effects in the task. The data file of 1 YA was corrupted and therefore, their data could not be used. Thus, we report data from 12 YA, 12 OA, and 10 PWA. The participants’ demographic information is presented in Table 1.

**Table 1:** Sample size, gender, years of age, and years of education for YA, OA, and PWA.

	***n***	**Gender (F:M)**	**Age (*M* [*SD*])**	**Education (*M* [*SD*])**
**YA**	12	7:5	20.6 [1.8]	14.8 [1.7]
**OA**	12	7:5	62.7 [9.33]	16.8 [2.3]
**PWA**	10	6:4	48.5 [14.3]	17.1 [5.5]

All participants were monolingual, native speakers of American English who reported normal or corrected-to-normal vision and hearing. All PWA presented with mild-to-moderate aphasia secondary to a left-hemisphere stroke and were between 20 and 38 months post-stroke at the time of participation in this study (*M* = 26.9, *SD* = 5.5). No participant reported a premorbid history of psychiatric or neurological conditions. Participants provided informed consent prior to study participation and were compensated upon completion of each session. This study was approved by the research ethics board at Purdue University.

### 2.2. Screening and language testing

All testing sessions were conducted *via* the videoconferencing platform, Cisco WebEx Meetings (Cisco WebEx, 2020), which was the preferred HIPAA-compliant platform at Purdue University at the time of testing. Prior to the study session, participants completed a pre-session Qualtrics survey (Qualtrics, 2020) which required them to check that they had access to high-speed internet (minimum speed of 1 Mbps download and upload), owned a computer or tablet with a screen larger than 13″ diagonally, and were able to use the videoconferencing software, Cisco WebEx Meetings (Cisco WebEx, 2020). They were also asked about their comfort level with using technology and any previous experience with internet-based research.

Informal hearing and vision screenings were conducted to verify that participants’ hearing and vision were at an acceptable level to complete tasks delivered over the internet. For the hearing screening, pure tones were created at 500, 1000, 2000, and 4,000 Hz at 40 dB so that the pitch was somewhat similar to an audiometer when a participant’s computer volume was set to 80%. Participants were asked to indicate whether they were able to hear the pure tones which were shared at 80% volume on their computer without headphones. Participants were required to confirm that their computer volume was set to 80% by sharing their screen through WebEx and to indicate that the volume level was comfortable before continuing. While we acknowledge the limitations of free-field audiometric screening, the purpose of the hearing screen was to ensure participants could hear the experimenter at a normal speech volume. All participants passed the hearing screening, indicating they could hear up to 2000 Hz at 40 dB. The vision screening contained objects that were adapted from Hyvärinen (2018) and placed into a PowerPoint presentation. The PowerPoint presentation contained two rows of objects: the top row showed the sequence of objects at the size that was being tested and one of these objects was highlighted in a red box. The bottom row showed another sequence of the objects at the maximum size (D 8 M). Macros were added to this PowerPoint so that the participant could select the objects and progress the slides themselves. Participants were asked to select the object in the bottom row that matched the object in the top row that was highlighted in the red box. After selecting the object, participants selected the “Next” button to move to the next object in the sequence. Participants were tested once at D 8 M (the largest object size) to ensure they understood the task and then tested at D 3.2 M, a size similar to that of the text in the stimuli for this study. All participants were able to see the objects at the D 3.2 M size.

To determine that all YA and OA exhibited normal cognition, the Mini-Mental State Examination (MMSE; Folstein et al., 1975) was used. The MMSE has been shown to be effective in screening for cognitive deficits when administered face-to-face and *via* videoconferencing (Carotenuto et al., 2018). All YA and OA scored above the established general criteria cutoff (> 24/30), indicating no abnormalities for age, education level, or risk of dementia (YA: *M* [*SD*] = 29.7 [0.7], *Mdn* = 30; OA: *M* [*SD*] = 28.9 [1.2], *Mdn* = 29). PWA were assessed using a combination of language tests, as shown in Table 2, in a 2-h long session prior to the study. The Western Aphasia Battery-Revised (WAB-R; Kertesz, 2006) was delivered first following the virtual testing procedures in Dekhtyar et al. (2020) and the guidelines of the WAB-R through Q-global (Pearson Assessments, 2020). Then, the Northwestern Assessment of Verbs and Sentences (NAVS; Thompson, 2011) was administered to assess comprehension and production of verbs and sentences in PWA. Finally, the Boston Naming Test (BNT 2nd Edition; Kaplan et al., 2001) was administered to assess noun retrieval abilities in PWA. The picture stimuli of the NAVS and the BNT were presented to participants *via* screen share. Some tests required participants to take control of the examiner’s computer to select objects or pictures. For PWA who needed technological assistance, their caregivers were present to assist with the initial setup of necessary tools for the session but were asked to leave the room once the session started.

**Table 2:** Language testing scores (with max scores) for PWA in Study 1 (TelePriming task).

	**WAB-R**					**NAVS**					**BNT**
**PWA**	**AQ (100)**	**Fluency (10)**	**AC (10)**	**Repetition** **(10)**	**Naming** **(10)**	**VNT (100)**	**VCT (100)**	**ASPT (100)**	**SPPT (100)**	**SCT (100)**	**Score** **(%)**
A1	94.1	9	9.5	8.8	9.8	91	100	100	100	70	97
A2	90.8	8	9.9	10	8.5	77	100	97	87	80	72
A3	75.4	5	8.3	7	8.4	59	100	94	43	60	82
A4	N/A	5	N/A	8.4	8.1	73	95	78	57	60	77
A5	62.5	5	7.3	4.6	6.4	82	100	78	25	63	55
A6	77.6	6	8.7	7.6	8.5	77	100	91	53	50	87
A7	65.3	4	8.9	6.6	6.1	59	100	63	27	50	52
A8	87.9	9	9.3	8	8.7	68	95	100	77	70	97
A9	81.3	8	8.9	6	8.7	95	100	100	33	70	77
A10	74.0	5	8.3	7.7	8.0	82	100	72	60	87	73
**Mean**	78.8	6.4	8.8	7.47	8.1	76	99	87	56	66	77
**SD**	10.9	1.9	0.8	1.52	1.1	12	2	14	25	12	15

WAB-R, Western Aphasia Battery–Revised; AQ, Aphasia Quotient; AC, Auditory Comprehension; NAVS, Northwestern Assessment of Verbs and Sentences; VNT, Verb Naming Test; VCT, Verb Comprehension Test; ASPT, Argument Structure Production Test; SPPT, Sentence Production Priming Test; SCT, Sentence Comprehension Test; BNT, Boston Naming Test.

We included PWA with mild-to-moderate aphasia given the experimental task required ability to comprehend sentence-level utterances and produce some nouns and verbs. All PWA had a diagnosis of aphasia following a L CVA, as verified by their medical records. On the WAB-R, they demonstrated the Aphasia Quotient (AQ) higher than 50/100, a fluency score 4 or greater, and naming score greater than 5/10. One PWA (A4) used a tablet instead of a laptop, which interfered with performance on the pointing tasks of the Auditory Comprehension (AC) of the WAB-R. Thus, we could not derive a total AC score and AQ for this participant (see Table 2 WAB scores for A4). On the NAVS, all PWA demonstrated relatively preserved comprehension of single verbs (95–100% Verb Comprehension Test) and 50% or higher accuracy in comprehension of varying sentence types (Sentence Comprehension Test). The scores from the Verb Naming Test and the Argument Structure Production Test indicated that the PWA were able to produce some verbs and sentence level utterances. All PWA showed 50% or higher accuracy on the Boston Naming Test (BNT). Although not assessed formally, informal observations during assessment indicated that no PWA had significant motor speech impairments.

### 2.3. Methods

#### 2.3.1. Experimental stimuli

The same stimuli used in Man et al. (2019) were used. The experimental stimuli consisted of a total of 48 prime and 48 target transitive sentences and their corresponding black-and-white line drawings. The original prime and target stimuli were taken from Branigan and McLean (2016) and were modified to include written words to minimize word retrieval difficulties for PWA in Man et al. (2019). The experimental stimuli depicted one of 6 transitive actions (bite, chase, kiss, lift, pull, push) with different animal agents and human themes (e.g., a dog is chasing a man; a horse is biting a robber). Additionally, a total of 96 intransitive filler items (e.g., the man is jumping) were included to separate experimental items. Fifteen of the filler pictures were used as “Bingo” items for the task of finding matching cards with the experimenter (see procedure for a more detailed description).

Two experimental lists were created, and each participant was tested on only one list. Each target picture was presented only once within the list with the prime type counterbalanced between the lists (e.g., if a prime sentence was active in list 1, then it would be a passive sentence in list 2). In addition, the same verb was used in half of the prime-target picture pairs (e.g., a horse pulling a girl – a tiger pulling a soldier), whereas different verbs were used in the other half prime-target picture pairs (a rabbit biting a queen – a dog chasing a man) to examine lexical boost. Within each list, the position of the agent appeared on the left side of the picture for half of the trials and on the right side for the other half.

#### 2.3.2. TelePriming task

**Figure 1:**
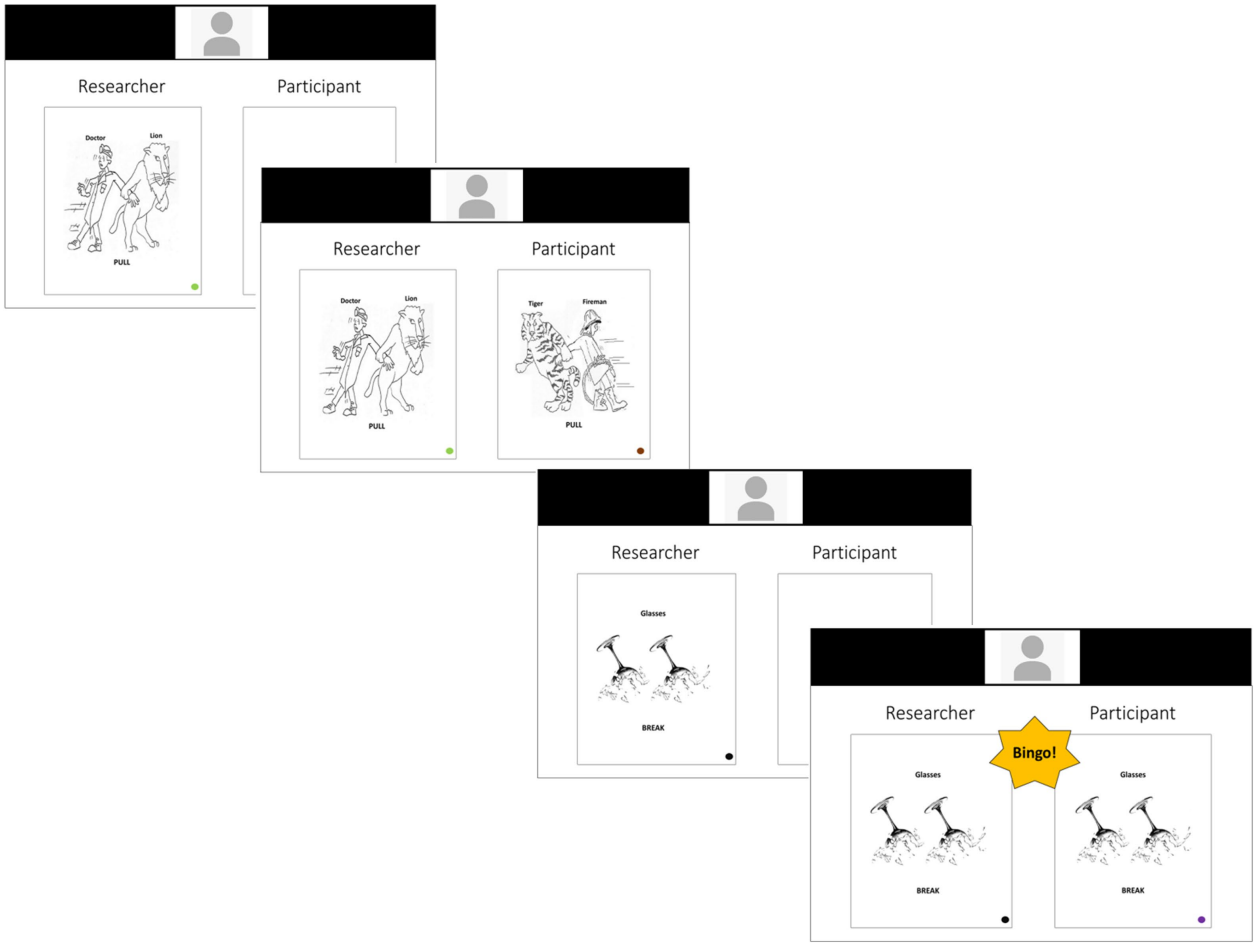
Example of the experimental layout in WebEx Meetings from the participant’s perspective.

For the TelePriming task, stimuli were presented in PowerPoint on the experimenter’s computer, which was shared with the participant *via* a secure videoconference platform, WebEx. The experimenter and participant took turns describing pictures using sentences in a picture matching game. Participants were told that the goal of the task was to determine whether their picture matched the experimenter’s picture. If the pictures matched, they were asked to say “Bingo!.” Figure 1 demonstrates the sequence of an experimental trial, including a prime-target pair and a matching (Bingo) filler pair. The experimenter progressed the PowerPoint to display the experimenter’s picture on the left and then the participant’s picture on the right. All pictures had a color cue in the bottom right corner. On the experimenter’s picture, the color cue indicated which sentence structure (‘green’ for active or ‘orange’ for passive) the experimenter should produce, and the color cue on the participant’s picture (gray or brown) was used to ensure that the layout of the pictures was kept consistent. The sentence structure that the experimenter produced when describing the picture on the left served as the prime sentence. We measured if participants were more likely to use the same sentence structure (active or passive) as the experimenter when it was their turn to describe their picture on the right. The experiment typically lasted 30 min for healthy adults and approximately an hour for PWA.

#### 2.3.3. Post-experiment survey

After the language testing and TelePriming task were completed, participants were asked to complete a post-session Qualtrics survey. The survey questions included ratings on the quality of the audio/video during the sessions, how comfortable participants were with using technology during the study, and whether they were willing to participate in future online research.

### 2.4. Data analysis

#### 2.4.1. Coding

Each target response was transcribed verbatim and coded as ‘correct’ or ‘incorrect’ responses. Only correct responses were used for the statistical analysis of priming effects. When self-corrected attempts were made, the final attempted sentential response was scored. A sentential response was defined as the production of at least the subject noun and a verb phrase.

Responses were considered ‘correct’ if they included all the target nouns and the verb in either an active (e.g., the dog chased the man) or a passive sentence structure (e.g., the man was chased by the dog). For both PWA and OA, variations in the verb tense (e.g., chase/chased/chasing) were accepted for active responses. However, only the past participle form of the main verb was accepted for passive responses, although variations of auxiliary verb were allowed (e.g., is/was chased by). For PWA, we allowed omission of auxiliary verbs (e.g., the man chased by the dog; the dog chasing the man), omission of articles (e.g., dog is chasing man), and intelligible phonological or speech sound errors (e.g., the dog is tasing the man). For incorrect responses produced by PWA, their error types were tallied. The specific error types and examples are provided in Table 3. No error analysis was conducted for YA and OA due to their high accuracies in production.

**Table 3:** Error types and example incorrect responses for PWA.

Error types	Examples (target picture: ‘a dog chases a man’)
*Role reversal*: thematic roles (agent, theme) of the nouns are reversed.	*The man is chasing the dog.* *The dog was chased by the man.*
*Argument omission*: when one of the required nouns is omitted	*The dog chasing.* *Chased by the dog.*
*Lexical*: Off-target noun or verb substitutions	*The cat is chasing the man.* *The dog is jumping the man.*
*Other:* non-sentential response; I do not know or abandoned responses; a response with multiple error types	*Dog and man* *The man…I do not know.* *The man is biting the dog.*

The primary dependent measure of the current study was the proportion of passive structures produced out of the correctly produced transitive (active and passive) sentences. Increased production of passive structures under the passive vs. active prime condition was considered to be a successful priming effect.

#### 2.4.2. Statistical analysis

Passive sentence production was modeled using mixed-effects logistic regression models with two within-subject factors—Verb (different or same) and Prime (passive or active)—and one between-subjects factor—Group (PWA or OA or YA). A subject-level random intercept and slope for the Verb effect were included. There was not a significant random slope for the Priming factor, so it was not included in the model. A main effects model was estimated along with models testing interactions between factors. The main effects model provides tests of differences in passive sentence production across the levels of the three independent factors controlling for the other independent factors. Two-way interactions involving Prime were evaluated in separate models to test hypotheses about differences in the Prime effect across the Group and Verb conditions. Finally, a full factorial model that included all two-way and three-way interactions among the three independent factors was estimated. This model tested for differences in the Prime effect across the Group-by-Verb combinations. Study participants provided between 23 to 48 trials each, resulting in a total sample of 1,494 observations (YA = 572, OA = 566, PWA = 356). Primary study hypotheses are within-subject effects, which are sufficiently powered given the repeated trials design and number of observations. Model estimates are reported on the logit scale, and model-predicted values are provided as percent passive correct. Stata v.17 was used to estimate models.

### 2.5. Results

#### 2.5.1. TelePriming results

On average, both young (YA) and older adults (OA) produced high proportions of total (both active and passive) correct sentences (99 and 98%, respectively). Persons with aphasia (PWA) produced correct sentences in 74% of the trials. Incorrect PWA responses contained role reversal errors (69%), argument omissions (6%), off-target lexical substitutions (19%), and other errors (6%).

The model testing main effects revealed a large prime effect, indicating that participants produced more passive sentences following passive vs. active primes, *b* = 3.71, *p* < 0.001. In addition, there was a Group effect such that OA generally produced more passive sentences compared to YA, *b* = 0.96, *p* = 0.036, and PWA produced fewer passive sentences than OA, *b* = −1.80, *p* < 0.001. However, there was no difference between PWA and YA, *b* = −0.84, *p* = 0.10. There was a Verb effect, indicating that passive sentence production was higher for the same verb condition compared to the different verb condition, *b* = 0.52, *p* = 0.005. Results for the main effects model are presented in Supplementary Table S1, and the percent of passive sentences produced is provided in Table 4.

**Table 4:** Predicted percentages from the main effects model.

	Percent passives	SE
PWA group	9.4	3.06
OA group	32.8	5.96
YA group	17.8	4.29
Different verb	16.4	2.74
Same verb	24.4	3.64
Active prime	4.6	1.12
Passive prime	54.1	4.10

*n* = 34, *o* = 1,494.

**Figure 2:**
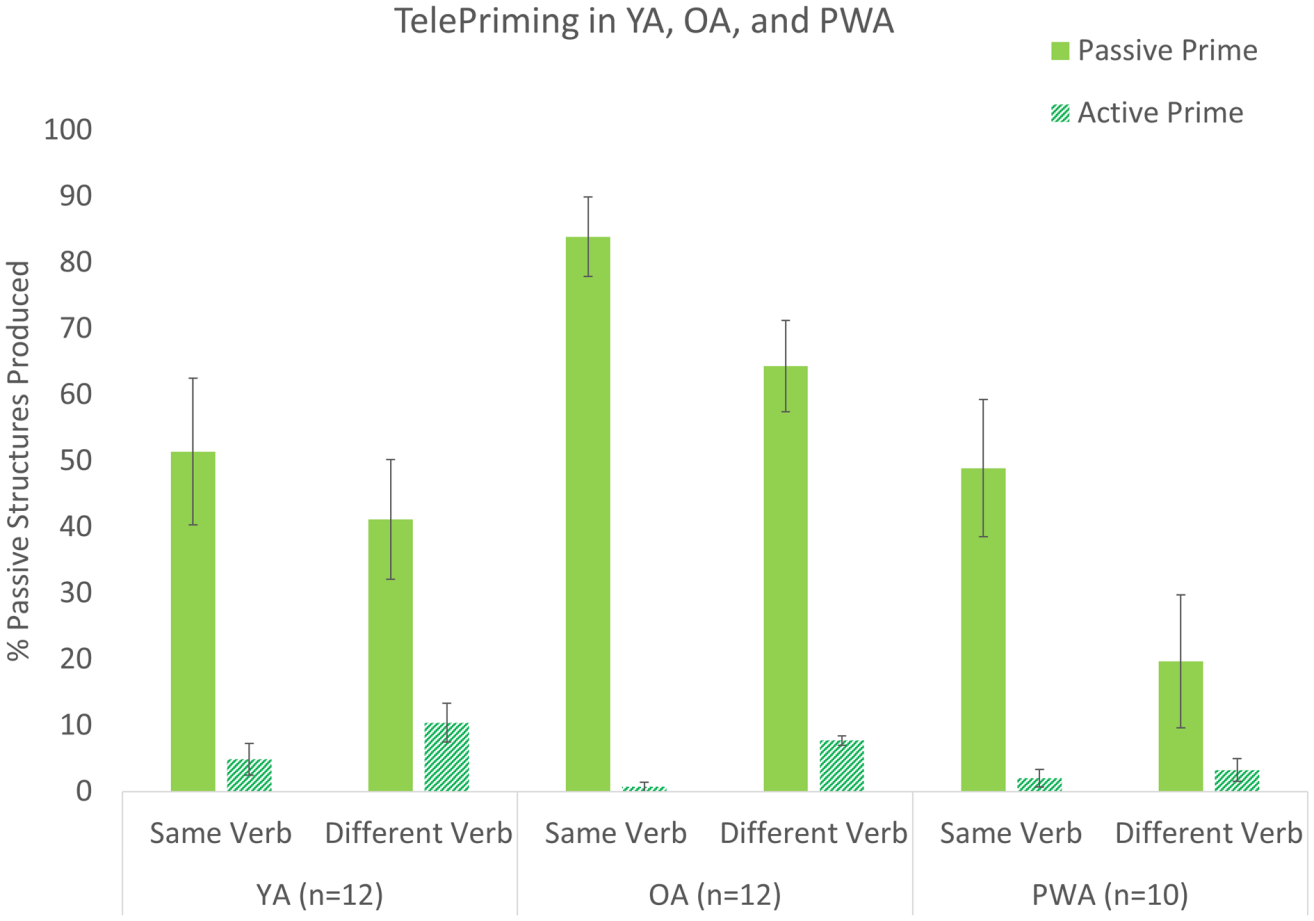
Priming effects for YA, OA, and PWA in Study 1 (TelePriming).

Tables 5 6 show the differences in percent of priming effects from the two-way and three-way interaction models. Figure 2 gives the predicted percentages from the three-way interaction model. Among the two-way interactions with the Prime condition, the interaction between Prime and Verb was significant, indicating a significant lexical boost effect, *b* = 2.45, *p* < 0.001 (see also Supplementary Table S2). That is, the effect of Prime was larger in the same verb condition (37.6%), compared to the different verb condition (61.3%) (see Table 5). In addition, there was a significant interaction between Prime and Group such that the prime effect differed between the OA group and the YA group, *b* = 1.94, *p* < 0.001 (Supplementary Table S3). However, there was no difference in the prime effect between the PWA and YA groups, *b* = 0.95, *p* = 0.109, and between the PWA and OA groups, *b* = −0.98, *p* = 0.120. The prime effect in the OA group was a difference of 68.5% while this difference was 39.4% for the YA group and 32.3% for the PWA group, as shown in Table 5.

**Table 5:** Simple effects of Priming from the two-way interaction models on the percent scale.

	Difference	Value of *p*	95% CI	
Passive v. Active for different verb	37.6	0.000	30.2	44.9
Passive v. Active for same Verb	61.3	0.000	52.7	69.9
Passive v. Active for PWA	32.3	0.000	19.1	45.6
Passive v. Active for OA	68.5	0.000	59.1	77.8
Passive v. Active for YA	39.4	0.000	29.2	49.7

*n* = 34, *o* = 1,494.

**Table 6:** Simple Prime effects from the three-way interaction model on the percent scale.

	Difference	Value of *p*	95% CI	
Passive v. Active: for PWA group and different verb	19.9	0.001	7.89	31.98
Passive v. Active: for OA group and different verb	54.7	0.000	43.51	65.92
Passive v. Active: for YA group and different verb	30.1	0.000	19.21	41.07
Passive v. Active: for PWA group and same verb	47.4	0.000	30.03	64.84
Passive v. Active: for OA group and same verb	82.8	0.000	72.80	92.78
Passive v. Active: for YA group and same verb	46.4	0.000	32.82	59.90

*n* = 34, *o* = 1,494.

Lastly, the three-way interaction effect between Group, Prime, and Verb was significant, such that there were differences in the Prime by Verb interaction (lexical boost effect) between the OA and YA groups, *b* = 2.54, *p* = 0.048. However, the lexical boost effect was not different between the PWA and YA groups, *b* = 0.34, *p* = 0.779, or between the PWA and OA groups, *b* = −2.20, *p* = 0.159 (see Supplementary Table S4). Post-hoc contrasts after the 3-way interaction indicated that the Prime effects were present in all three groups within verb condition, as shown in Table 6. In addition, the Prime by Verb interaction was significant in OA and YA, and approached significance in PWA. Specifically, the YA group, on average, produced 46% more passive sentences after a passive versus active prime in the same verb condition, whereas the average priming effect was 30% in the different verb condition (*p* = 0.004). OA showed on average 83% vs. 55% priming effects in the same vs. different verb conditions (*p* < 0.001). PWA showed 47 and 20% differences in passive production in the same vs. different verb conditions, respectively (*p* = 0.057).

#### 2.5.2. Survey results

**Figure 3:**
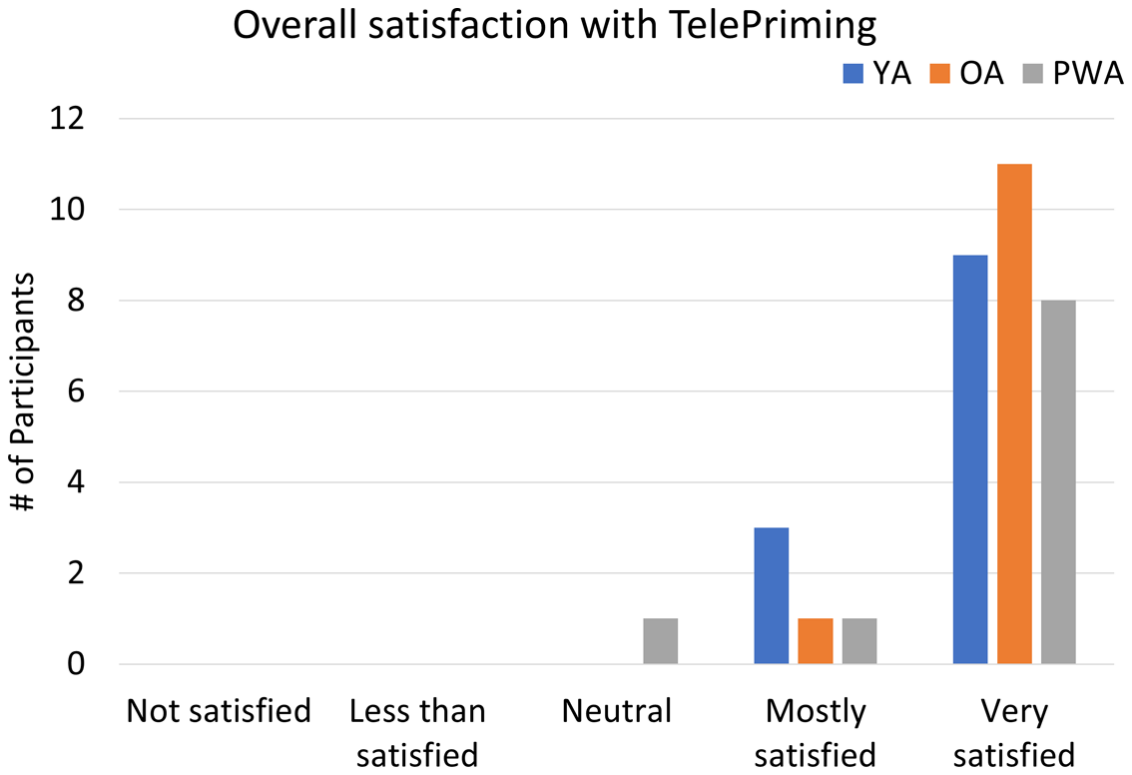
Survey results for overall satisfaction with the TelePriming task for YA, OA, and PWA.

The post-session survey results revealed that 75% (9/12) of YA participants were ‘very satisfied’ with the TelePriming study. In addition, 92% (11/12) of OA and 80% (8/10) of PWA reported being ‘very satisfied’ with TelePriming (see Figure 3).

**Figure 4:**
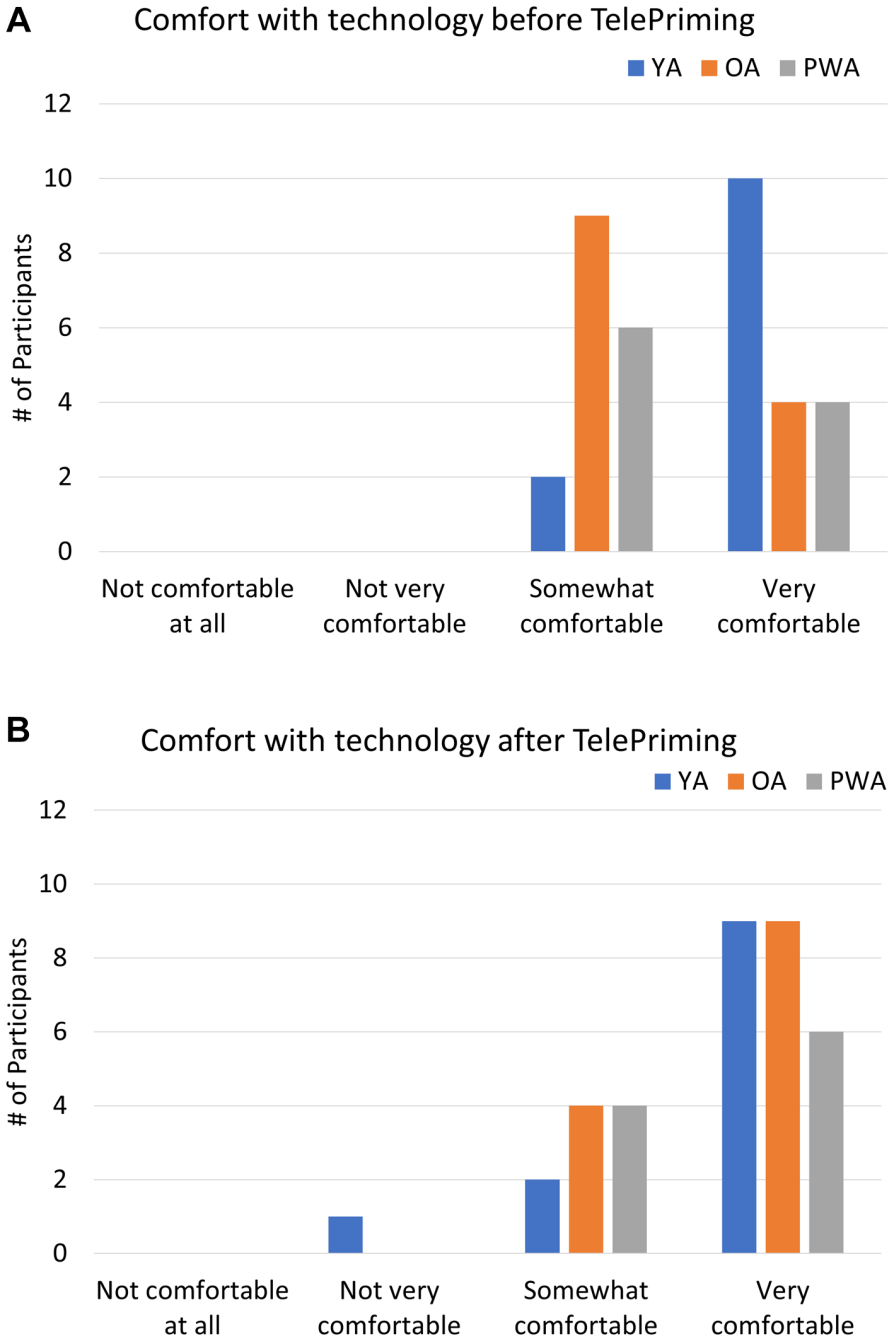
Survey results on the participant’s level of comfort with using technology for research before **(A)** and after **(B)** the TelePriming task for YA, OA, and PWA.

With respect to participants’ comfort level with using technology before and after the TelePriming study, the results indicated that there was an increase in the number of OA and PWA participants who felt ‘very comfortable’ after TelePriming compared to being ‘somewhat comfortable’ before the study. YA did not show a notable change in comfort level with technology after the study, although one participant reported being ‘not very comfortable’ due to connectivity problems (see Figure 4).

## 3. Study 2

In Study 2, we compared the data of the OA and PWA in Study 1 (TelePriming) to the data that were collected from our previous in-person dialogue-like priming study (Farr, 2020) to examine whether TelePriming resulted in comparable priming effects for PWA and OA. The in-person priming task was identical to the TelePriming task in terms of the experimental design and stimuli, except for the mode of testing.

### 3.1. Participants

Twelve PWA and 12 OA participated in the in-person priming task. None of these participants took part in the Telepriming task in Study 1, except for one PWA who completed both in-person and TelePrming tasks, 1 year apart. All participants were monolingual, native American English speakers with no reported history of neurological or psychological disorders prior to stroke that would affect their communication. Demographic data for the OA and PWA groups are shown in Table 7. All participants passed a hearing screening at 500, 1000, and 2000 Hz at 40 dB in at least one ear. OA were assessed on their cognitive-linguistics abilities by completing the Cognitive Linguistic Quick Test (CLQT; Helm-Estabrooks, 2001) and scored within normal limits for their age range based on the Clinical Severity Rating (3.8–4.0/4.0). The participants for this study were tested in person at Purdue University.

**Table 7:** Demographic information for OA and PWA in the in-person priming task.

	***n***	**Gender (F:M)**	**Age (*M* [*SD*])**	**Education (*M* [*SD*])**
**OA**	12	9:3	71.5 [6.3]	15.8 [2.3]
**PWA**	12	7:5	68.5 [14.0]	14 [2.0]

All PWA had a diagnosis of aphasia following a left CVA at least 6 months prior to their participation in the experiment. The same battery of tests as Study 1 was administered to PWA. The results of these tests are presented in Table 8.

**Table 8:** Language test scores (with max scores) for PWA in Study 2 (In-person priming task).

	**WAB-R**					**NAVS**					**BNT**
**PWA**	**AQ (100)**	**Fluency (10)**	**AC (10)**	**Repetition (10)**	**Naming (10)**	**VNT (100)**	**VCT (100)**	**ASPT (100)**	**SPPT (100)**	**SCT (100)**	**Score (%)**
A1	69.6	5	8.7	4.4	7.7	73	100	100	7	60	73
A2	77	6	8.8	7.4	7.3	50	100	94	43	70	58
A3	70.3	8	5.3	6	6.9	80	100	69	7	47	35
A4	93.1	9	9.9	8.6	9.1	95	100	100	100	100	87
A5	85	8	9.3	9.5	6.7	95	100	100	87	77	62
A6	87.7	8	9.6	8.4	8.9	83	100	94	84	90	63
A7	96.2	9	10	9.4	9.7	100	100	100	100	90	100
A8	75.2	6	8.5	6.7	8.4	82	95	69	27	57	68
A9	96.2	9	10	9.4	9.7	100	100	100	100	100	97
A10	94.4	9	9.2	9	10	100	100	100	97	100	97
A11	73	4	8.6	10	6	14	91	56	57	83	28
A12	84.6	6	9.1	9	9.2	100	100	97	87	97	98
Mean	83.5	7.3	8.9	8.2	8.3	81	99	90	66	81	72
SD	10.2	1.8	1.3	1.7	1.3	26	3	16	37	19	24

WAB-R, Western Aphasia Battery–Revised; AQ, Aphasia Quotient; AC, Auditory Comprehension; NAVS, Northwestern Assessment of Verbs and Sentences; VNT, Verb Naming Test; VCT, Verb Comprehension Test; ASPT, Argument Structure Production Test; SPPT, Sentence Production Priming Test; SCT, Sentence Comprehension Test; BNT, Boston Naming Test.

Similar to the participants in the TelePriming task of Study 1, the PWA who completed the in-person priming task presented with mild-to-moderate aphasia. PWA demonstrated relatively preserved comprehension of single words and sentences, as indicated by the scores on the Verb Comprehension Test (VCT), and above chance-level performance on the Sentence Comprehension Test (SCT). We also ensured that the participants could produce some single words and simple sentences as measured by the Naming subtest of the WAB-R and Verb Naming and Argument Structure Production Tests (VNT, ASPT) of the NAVS. Importantly, the PWA who completed the in-person priming task did not statistically differ from the PWA of Study 1 on all language measures, *t*(20) < 2.3, *p*s > 0.05, except for the Sentence Comprehension Test of the NAVS, *t*(20) = 2.18, *p* = 0.041. PWA in the in-person priming task showed higher comprehension scores on the SCT of the NAVS than the PWA who completed the TelePriming task.

### 3.2. Statistical analysis

Passive sentence production was modeled using mixed-effects logistic regression models with two within-subject factors—Verb (different or same) and Prime (passive or active)—along with one between-subjects factor—Study Type (Tele or In-person)—and one between-subject control variable: Age. Age was included as a between-subject control variable, because age was different between the study groups for OA and PWA, and some research has shown that age could interact with priming effects (e.g., Heyselaar et al., 2021). A subject-level random intercept and random slope for the Prime effect was included. There was not a significant random slope for the Verb effect, so it was not included in the models. Models were estimated separately for the OA and PWA groups. A main effects model was estimated along with models testing interactions between the experimental factors of Prime, Verb, and Study Type. Finally, a full factorial model that included all two-way and three-way interactions among Prime, Verb, and Study was estimated. The groups were also pooled to determine whether the Group effects differed across the Study Type. Because the years of education and SCT scores of the NAVS differed across the tele- and in-person PWA groups, a sensitivity analysis for the PWA group was undertaken where years of education and the SCT scores were included in the models as a control and interacted with study type. Aphasia severity as measured by the AQ was also considered in the sensitivity analysis. OA group subjects (*n* = 24) provide 1,132 observations and PWA subjects (*n* = 21) provide 872 observations. Primary study hypotheses are within-subject effects, which are sufficiently powered given the repeated trials design and number of observations. Model estimates are reported on the logit scale and model-predicted values are provided as percent passive correct. Stata v.17 was used to estimate models.

### 3.3. Results

The older adults (OA) in the in-person priming study produced correct responses in 98% of experimental trials, similar to the OA in the TelePriming task. PWA produced codable sentences in 92% of the trials. Incorrect PWA responses contained role reversal errors (70%), incorrect argument structures (4%), lexical substitutions (12%), and combinations of the preceding errors (14%).

**Figure 5:**
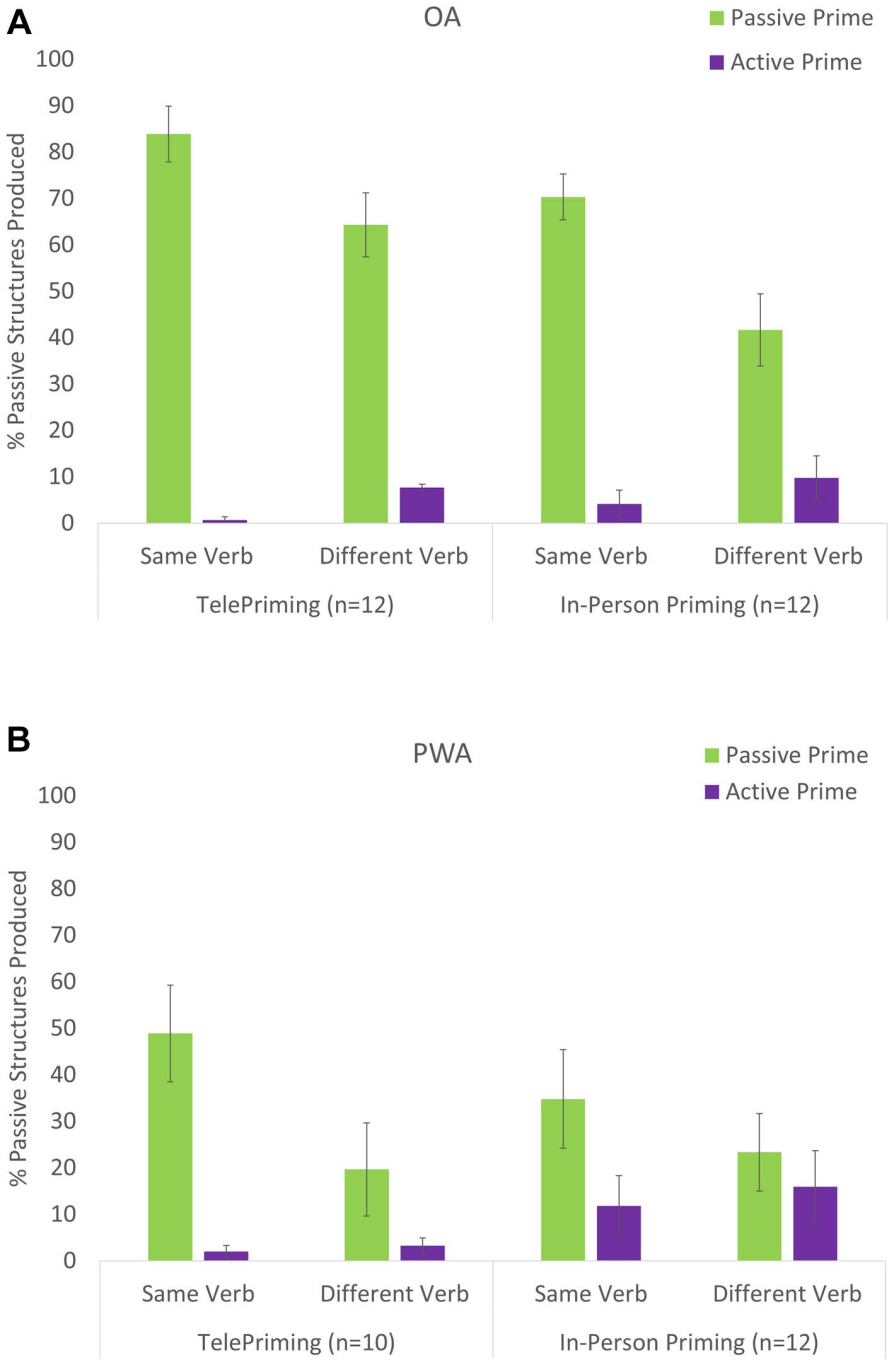
Comparisons of the priming effects in OA **(A)** and PWA **(B)** for the TelePriming (Study 1) and In-person (Study 2) priming tasks.

There was a statistically significant and large priming effect for both the OA, *b* = 4.12, *p* < 0.001, and PWA, *b* = 2.13, *p* < 0.001 groups when controlling for Study Type. However, there was no difference in the outcome (percent correct) by Study Type in either group, *b* = 0.28, *p* = 0.635 for OA and *b* = 0.68, *p* = 0.264 (Supplementary Tables S5, S6). The Prime by Verb interaction found in Study 1 was replicated across Study Types within Groups in Study 2 (*b* = 2.81, *p* < 0.001 for OA and *b* = 1.94, *p* < 0.001 for PWA). There was no difference in Group or Verb condition effects across the tele- and in-person Study Types. However, the priming effect was larger in the tele study compared to the in-person study for both the OA group (*b* = 1.39, *p* = 0.033) and PWA group (*b* = 1.77, *p* = 0.051). The percent differences are shown in Table 9. There was not a Prime by Verb by Study Type three-way interaction within Groups (*b* = 1.44, *p* = 0.251 for OA and *b* = 0.75, *p* = 0.535 for PWA) That is, both OA and PWA groups showed similar lexical boost effects between the in-person and TelePriming tasks. The sensitivity analysis showed that the results for the PWA group did not change after controlling for education, WAB AQ, and SCT scores. In addition, neither the WAB AQ nor SCT scores of the NAVS interacted with the Study Type for PWA. Figure 5 portrays the percentages from the three-way interaction models and demonstrates the priming effect differences across Study Types for each Group and the differences by Verb condition.

**Table 9:** Simple Prime effects from the two-way interaction models on the percent scale.

	Difference	Value of *p*	95% CI	
OA Group Model *n* = 21, *o* = 1,132				
Passive v. Active for In-Person Study	48.8	0.000	34.2	63.4
Passive v. Active for Tele Study	69.9	0.000	57.3	82.4
PWA Group Model *n* = 21, *o* = 872				
Passive v. Active for In-Person Study	14.6	0.008	3.7	25.5
Passive v. Active for Tele Study	29.6	0.000	15.3	44.0

## 4. Discussion

Application of videoconferencing to the assessment and treatment of aphasia has been increasing rapidly since COVID-19. Telepractice provides many potential benefits, including improved accessibility of cost-effective speech services and research sessions for persons with aphasia (PWA). However, there is still a need to develop more treatments targeting sentence production in PWA that can be delivered through videoconferencing. Structural priming has received recent attention as a potential training method for PWA, although the research is still at an early stage. The current study examined whether TelePriming in a dialogue-like structural priming task would be as effective as in-person priming in eliciting production of passive sentences in PWA.

### 4.1. Effectiveness of TelePriming

The main findings of Study 1 support the robust effectiveness of TelePriming in all three groups of young adults (YA), older adults (OA), and PWA. When describing transitive events, participants showed a strong tendency to re-use the same sentence structure as the experimenter to describe their picture, thus producing more passive structures after they heard passive prime sentences compared to active prime sentences (see Figure 2). The young adults showed 46 and 30% priming effects in the same and different verb conditions, respectively, replicating previous studies which used a similar collaborative priming task in a laboratory setting (e.g., Branigan et al., 2000; Cleland and Pickering, 2003; Branigan and McLean, 2016). The older adults showed 82 and 54% priming effects in the same vs. different verb conditions, which were significantly larger than the priming effects than in the young adults. The larger priming effects seen in the older adults are interesting and could be due to an inverse priming effect where priming is increased due to the unfamiliarity with the structure (Chang et al., 2006; Jaeger and Snider, 2013). Older adults use syntactically complex sentences less frequently than young adults (e.g., Kemper, 1987; Kemper et al., 2004). Therefore, hearing their interlocutor’s production of passives might have created a greater tendency for priming (see also Lee et al., 2022 for similar findings).

Most importantly, PWA showed as large of a priming effect as young adults, resulting in 47 and 20% increases in passive sentence production in the same and different verb conditions, respectively. The PWA group showed a smaller priming effect than the OA group, different from the inverse priming effect seen between the OA and YA groups. However, previous studies also found reduced priming effects for PWA compared OA, particularly when they used comprehension-to-production priming tasks (Lee et al., 2019; Man et al., 2019; van Boxtel et al., 2023). With their impaired language processing, encoding and activation of prime sentences might not have been strong enough in our PWA, when they did not have to overtly produce prime sentences. Or it could simply be because the OA in Study 1 showed particularly larger priming effects. Nonetheless, given that our PWA were not explicitly instructed to pay attention to their interlocutor’s sentences or to produce the same sentence forms and since difficulty producing complex sentences such as passives is pervasive in PWA, the robust faciliatory effects seen in the PWA are fascinating. Despite their language deficits, after simply hearing their interlocutors’ production of different sentences, PWA were able re-use those structures in their own production. Overall, the main findings of Study 1 are consistent with previous findings from laboratory-based settings, where similar collaborative priming tasks were used in older adults (Hardy et al., 2017; Lee et al., 2022) and in persons with aphasia (Lee et al., 2019; Man et al., 2019).

Study 2 further confirmed the effectiveness of TelePriming in older adults and PWA. Participant-specific differences between the TelePriming and the in-person priming task, including differences in age (for both groups) and aphasia severity (as measured by the WAB AQ), and the sentence comprehension abilities of PWA did not significantly interact with the mode of the priming task. In fact, although unexpected, both OA and PWA showed larger priming effects in the TelePriming compared to the in-person priming task. It is unclear why the TelePriming task elicited greater priming effects in the participants. One possible reason is that unfamiliarity with technology encouraged the older adults and PWA to become more attentive to the prime sentences produced by the examiner, generating increased priming effects. The findings from Study 1 and 2 clearly suggest that collaborative priming tasks, specifically scripted dialogue tasks are effective in both older adults and PWA, regardless of the mode of task delivery.

### 4.2. Survey results

The results of the pre- and post-session surveys from Study 1 showed that the TelePriming task used in the current study is feasible and appropriate for our PWA and older adults. Even when participants were not maximally comfortable or satisfied with internet-based research, they were still willing to participate in internet-based research in the future and were generally satisfied with how the session progressed. In addition, both OA and PWA indicated increased comfort with the technology used after the session ended compared to before the session, although YA did not show a notable change in comfort level with technology. The survey results from our participants further suggest that TelePriming is feasible and are in line with previous telepractice studies where relatively high satisfaction with remote sessions was reported by PWA and clinicians (Pitt et al., 2017; Dekhtyar et al., 2020; Øra et al., 2020).

### 4.3. Effects of verb overlap

The last question of this study was whether participants would show a significant lexical boost effect when verbs were repeated between the prime and target sentences. In the TelePriming task, all three groups of participants showed a significant lexical boost effect, demonstrating increased priming effects when the verb overlapped between prime and target. Although the effect was borderline significant for PWA, the average 20% increase seen in our PWA in the same verb condition is quite large and is comparable with the lexical boost effect (16% boost) seen in the young adult group. Similarly, a lexical boost effect was also found in the in-person priming task in Study 2 for both groups, although they were numerically smaller than those seen in the TelePriming task. The significantly enhanced priming effects in the same vs. different verb priming conditions are consistent with predictions from the Interactive Alignment Theory (Pickering and Garrod, 2004, 2007). Interactive communication tasks, such as dialogue, facilitate spreading activation across multiple levels of linguistic information, yielding greater syntactic alignment among speakers (measured in priming effects) when both syntactic and lexical information are shared between what was heard and what is to be described, compared to when only syntactic structure is shared. Having lexical (verb) overlap between prime and target PWA might have ameliorated some of the lexical retrieval deficits in PWA, which in turn increased spreading activation from the lemma to its associated syntactic structures.

Different from the current study, Man et al. (2019) only found a significant lexical boost effect in OA but not in PWA, although the same scripted dialogue task was used. However, in the experimental design of Man et al. (2019), both transitive and dative stimuli were elicited in a mixed order. Therefore, Man et al.’s task was twice as long and involved processing multiple structural alternations, as opposed to the current study which only used the transitive stimuli. It is possible that the lexical information from the prime sentences that participants heard was not maintained strong enough in their PWA to exert additional lexical boost in structural priming. Studies utilizing a dialogue-like priming task may include lexical repetition to facilitate stronger access and use of certain syntactic structures in PWA. Having lexical overlap might ameliorate lexical encoding and retrieval difficulties that are common in PWA, which in turn eases the encoding and retrieval of the associated target structures. However, given that the current results are limited to immediate priming effects, further research is necessary to determine whether lexical repetition is a critical factor for maximal outcomes when a dialogue-like priming task is used as a multi-session treatment for PWA.

### 4.4. General discussion

This is the first study to demonstrate the feasibility and validity of an internet-based, dialogue-like priming task in older adults and persons with aphasia. Even when the collaborative priming task was delivered remotely, the two interlocutors still jointly worked to achieve a shared communicative goal, which in turn allowed the processes of production and comprehension to become tightly coupled in a manner that created implicit priming of linguistic representations (Pickering and Garrod, 2004, 2007). The current results provide strong empirical bases for researchers and clinicians to implement structural priming tasks using videoconferencing with aging and clinical populations and to expect at least comparable or greater results as in-person priming tasks.

Additionally, the current findings suggest that the alignment process of message-sentence structure mappings in an interactive language task remains preserved in aphasia, extending the burgeoning evidence of the positive effects of structural priming in PWA (for production: Saffran and Martin, 1997; Hartsuiker and Kolk, 1998; Cho-Reyes et al., 2016; Yan et al., 2018; Lee et al., 2019; Man et al., 2019; van Boxtel et al., 2023; for comprehension: Lee et al., 2019; Keen and Lee, 2022). Implicit structural priming is effective in facilitating sentence processing in PWA across different linguistic representations (syntactic, lexical), task types (monologue, dialogue), and delivery modes (in-person, TelePriming). These discoveries offer researchers and clinicians flexible ways to apply structural priming to PWA to assess their ability to activate certain linguistic representations under implicit priming or as an intervention strategy to ameliorate impaired message-structure mapping processes in PWA (Lee and Man, 2017; Lee et al., 2023).

Lastly, TelePriming may be more easily adapted to teletherapy than currently available sentence production treatments of aphasia. One of the most difficult barriers for persons with aphasia is mobility and access to therapy. Although sentence production deficits are pervasive in PWA at different stages of recovery, there is a paucity of sentence production treatments that can be easily delivered over videoconferencing. Structural priming paradigms such as the one used in this study require minimal to no manipulation of physical stimuli and provide simple instructions to follow and can thus be easily adapted for remote delivery. Mobility would no longer be an issue, as the clinician and patient could meet directly through a videoconferencing platform and have access to all of the materials in real-time. This may also reduce the cost of therapy, as internet-based therapy eliminates the cost of transportation to therapy sessions. Since this study established the validity of delivering structural priming over the internet at an early proof-of-concept experimental testing phase, future studies should replicate the effects of TelePriming over multiple training sessions to establish stronger efficacy data.

In conclusion, we demonstrated the feasibility and validity of an internet-based dialogue-like structural priming task. Persons with aphasia, young adults, and older adults all exhibited significant priming effects in a collaborative priming task delivered over videoconferencing, i.e., TelePriming. In addition, older adults and PWA showed larger structural priming effects in the TelePriming compared to in-person priming task. These findings suggest that interactive message-structure alignment processes remain largely intact in PWA and that the positive effects of structural priming are not diminished by remote delivery. These findings lay the experimental groundwork for future research, where TelePriming can be used for the assessment and treatment of sentence production deficits of PWA with limited access to face-to-face sessions.

## Data availability statement

The datasets presented in this study can be found in online repositories. The names of the repository/repositories and accession number(s) can be found below: https://osf.io/2rwhf/.

## Ethics statement

The studies involving humans were approved by Purdue Human Research Protection Program and Institutional Review Board. The studies were conducted in accordance with the local legislation and institutional requirements. The participants provided their written informed consent to participate in this study.

## Author contributions

JL: Conceptualization, Funding acquisition, Methodology, Supervision, Writing – original draft, Writing – review & editing. AK: Conceptualization, Data curation, Investigation, Methodology, Project administration, Visualization, Writing – original draft, Writing – review & editing. EF: Conceptualization, Data curation, Investigation, Methodology, Project administration, Writing – original draft. SC: Formal analysis, Visualization, Writing – original draft.

## References

[ref1] Agostini M. Garzon M. Benavides-Varela S. De Pellegrin S. Bencini G. Rossi G.. (2014). Telerehabilitation in poststroke anomia. Biomed. Res. Int. 2014:706909. doi: 10.1155/2014/706909, PMID: 24829914 PMC4009336

[ref1001] Allen M. L. Haywood S. Rajendran G. Branigan H. (2011). Evidence for syntactic alignment in children with autism. Dev. Sci. 14, 540–548. doi: 10.1111/j.1467-7687.2010.01001.x 21477193

[ref2] Bahadir G. Polinsky M. (2012). Is structural priming sensitive to the phrase-clause distinction? Concealed question NPs versus embedded interrogatives and declaratives. CogSci. Available at: https://scholar.harvard.edu/files/gbahadir/files/bahadirpolinsky_2012_paper.pdf

[ref3] Bock J. K. (1986). Syntactic persistence in language production. Cogn. Psychol. 18, 355–387. doi: 10.1016/0010-0285(86)90004-6, PMID: 36856897

[ref4] Bock K. Dell G. S. Chang F. Onishi K. H. (2007). Persistent structural priming from language comprehension to language production. Cognition 104, 437–458. doi: 10.1016/j.cognition.2006.07.003, PMID: 16973143

[ref5] Bock K. Griffin Z. M. (2000). The persistence of structural priming: transient activation or implicit learning? J. Exp. Psychol. Gen. 129, 177–192. doi: 10.1037/0096-3445.129.2.177, PMID: 10868333

[ref6] Brady M. C. Kelly H. Godwin J. Enderby P. Campbell P. (2016). Speech and language therapy for aphasia following stroke. Cochrane Database Syst. Rev. 2016:CD000425. doi: 10.1002/14651858.CD000425.pub4, PMID: 27245310 PMC8078645

[ref7] Braley M. Sims Pierce J. Saxena S. De Oliveira E. Taraboanta L. Anantha V.. (2021). A virtual, randomized, control trial of a digital therapeutic for speech, language, and cognitive intervention in post-stroke persons with aphasia. Front. Neurol. 12:626780. doi: 10.3389/fneur.2021.626780, PMID: 33643204 PMC7907641

[ref8] Branigan H. P. McLean J. F. (2016). What children learn from adults’ utterances: an ephemeral lexical boost and persistent syntactic priming in adult–child dialogue. J. Mem. Lang. 91, 141–157. doi: 10.1016/j.jml.2016.02.002

[ref9] Branigan H. P. Pickering M. J. Cleland A. A. (2000). Syntactic co-ordination in dialogue. Cognition 75, B13–B25. doi: 10.1016/S0010-0277(99)00081-5, PMID: 10771277

[ref10] Branigan H. P. Pickering M. J. McLean J. F. Cleland A. A. (2007). Syntactic alignment and participant role in dialogue. Cognition 104, 163–197. doi: 10.1016/j.cognition.2006.05.006, PMID: 16876778

[ref11] Carotenuto A. Rea R. Traini E. Ricci G. Fasanaro A. M. Amenta F. (2018). Cognitive assessment of patiens with Alzheimer’s disease by telemedicine: pilot study. JMIR Mental Health 5, 1–9. doi: 10.2196/mental.8097 PMC5970283 29752254

[ref12] Chang F. Dell G. S. Bock K. (2006). Becoming syntactic. Psychol. Rev. 113, 234–272. doi: 10.1037/0033-295X.113.2.234, PMID: 16637761

[ref13] Chang F. Janciauskas M. Fitz H. (2012). Language adaptation and learning: getting explicit about implicit learning. Lang. Linguist. Compass. 6, 259–278. doi: 10.1002/lnc3.337

[ref14] Cho-Reyes S. Mack J. E. Thompson C. K. (2016). Grammatical encoding and learning in agrammatic aphasia: evidence from structural priming. J. Mem. Lang. 91, 202–218. doi: 10.1016/j.jml.2016.02.004, PMID: 28924328 PMC5600488

[ref15] Cisco WebEx. (2020). WebEx meetings (version 40.10.10.22) [computer software]. Available at: https://www.webex.com/download

[ref16] Cleland A. A. Pickering M. J. (2003). The use of lexical and syntactic information in language production: evidence from the priming of noun-phrase structure. J. Mem. Lang. 49, 214–230. doi: 10.1016/S0749-596X(03)00060-3

[ref17] Coleman L. Hale T. M. Cotton S. R. Gibson P. (2015). The impact of information and communication technology (ICT) usage on psychological well-being among urban youth. Sociologic. Stud. Child. Youth 19, 267–291. doi: 10.1108/S1537-466120150000019008

[ref18] Corley M. Scheepers C. (2002). Syntactic priming in English sentence production: categorical and latency evidence from an internet-based study. Psychon. Bull. Rev. 9, 126–131. doi: 10.3758/BF03196267, PMID: 12026944

[ref19] Dechêne L. Tousignant M. Boissy P. Macoir J. Héroux S. Hamel M.. (2011). Simulated in-home teletreatment for anomia. Int. J. Telerehabilitation. 3, 3–10. doi: 10.5195/IJT.2011.6075, PMID: 25945183 PMC4296805

[ref20] Dekhtyar M. Braun E. J. Billot A. Foo L. Kiran S. (2020). Videoconference administration of the Western aphasia battery – revised: feasibility and validity. Am. J. Speech Lang. Pathol. 29, 673–687. doi: 10.1044/2019_AJSLP-19-00023, PMID: 32191122 PMC7842871

[ref21] Dial H. R. Hinshelwood H. A. Grasso S. M. Hubbard H. I. Gorno-Tempini M.-L. Henry M. L. (2019). Investigating the utility of teletherapy in individuals with primary progressive aphasia. Clin. Interv. Aging 2019, 452–471. doi: 10.2147/CIA.S178878 PMC6394239 30880927

[ref22] Farr E. (2020). Structural priming in aphasia using a blocked stimulus design (order no. 30503626). Available from Dissertations & Theses @ big ten academic Alliance; ProQuest one academic (2827706799).

[ref24] Folstein M. F. Folstein S. E. McHugh P. R. (1975). Mini-mental state: a practical method for grading the cognitive state of patients for the clinician. J. Psychiatr. Res. 12, 189–198. doi: 10.1016/0022-3956(75)90026-6, PMID: 1202204

[ref25] Fridler N. Rosen K. Menahemi-Falkov M. Herzberg O. Lev A. Kaplan D.. (2012). Tele-rehabilitation therapy vs. face-to-face therapy for aphasic patients. In Proceeding of International conference on eHealth, telemedicine, and social medicine.

[ref26] Furnas D. W. Edmonds L. A. (2014). The effect of computerized verb network strengthening treatment on lexical retrieval in aphasia. Aphasiology 28, 401–420. doi: 10.1080/02687038.2013.869304 PMC2744980 19763227

[ref27] Garraffa M. Coco M. Branigan H. (2015). Effects of immediate and cumulative syntactic experience in language impairment: evidence from priming of subject relatives in children with SLI. Lang. Learn. Develop. 11, 18–40. doi: 10.1080/15475441.2013.876277

[ref28] Garraffa M. Smith G. (2022). “Syntactic priming as a window to investigate grammatical learning in non-typical populations,” in Syntactic priming in language acquisition: representations, mechanisms and applications. ed. Messenger K. (Trends in Language Acquisition Research) John Benjamins. 31, 183–201.

[ref29] Getty D. J. Fraundorf S. H. (2023). Phrase-by-phrase self-pacing tasks are not sensitive to structural priming: methodological and theoretical implications. PsyArXiv. doi: 10.31234/osf.io/ytdb2

[ref30] Goldberg S. E. Whittamore K. H. Harwood R. H. Bradshaw L. E. Gladman J. R. Jones R. G. (2012). The prevalence of mental health problems among older adults admitted as an emergency to a general hospital. Age Ageing 41, 80–86. doi: 10.1093/ageing/afr106, PMID: 21890483 PMC3234074

[ref31] Gruberg N. Ostrand R. Momma S. Ferreira V. S. (2019). Syntactic entrainment: the repetition of syntactic structures in event descriptions. J. Mem. Lang. 107, 216–232. doi: 10.1016/j.jml.2019.04.005, PMID: 31942088 PMC6961959

[ref32] Hall N. Boisvert M. Steele R. (2013). Telepractice in the assessment and treatment of individuals with aphasia: a systematic review. Int. J. Telerehabilitation 5, 27–38. doi: 10.5195/ijt.2013.6119, PMID: 25945211 PMC4296832

[ref33] Hardy S. M. Messenger K. Maylor E. A. (2017). Aging and syntactic representations: evidence of preserved syntactic priming and lexical boost. Psychol. Aging 32, 588–596. doi: 10.1037/pag0000180, PMID: 28891670

[ref34] Hartsuiker R. J. Bernolet S. Schoonbaert S. Speybroeck S. Anderelst D. (2008). Syntactic priming persists while the lexical boost decays: evidence from written and spoken dialogue. J. Mem. Lang. 58, 214–238. doi: 10.1016/j.jml.2007.07.003

[ref35] Hartsuiker R. J. Kolk H. H. (1998). Syntactic facilitation in agrammatic sentence production. Brain Lang. 62, 221–254. doi: 10.1006/brln.1997.1905, PMID: 9576823

[ref36] Hartsuiker R. J. Westenberg C. (2000). Word order priming in written and spoken sentence production. Cognition 75, B27–B39. doi: 10.1016/S0010-0277(99)00080-3, PMID: 10771278

[ref37] Helm-Estabrooks N. (2001). Cognitive linguistic quick test: CLQT. San Antonio, TX: Psychological Corporation.

[ref38] Heyselaar E. Wheeldon L. Segaert K. (2021). Structural priming is supported by different components of nondeclarative memory: evidence from priming across the lifespan. J. Exp. Psychol. Learn. Mem. Cogn. 47, 820–837. doi: 10.1037/xlm0000955, PMID: 33151717

[ref39] Hill A. Theodoros D. G. Russell T. Ward E. C. (2009). The effects of aphasia severity on the ability to assess language disorders via telerehabilitation. Aphasiology 23, 627–642. doi: 10.1080/02687030801909659

[ref40] Hyvärinen L. (2018). Lea symbol near vision screener card [measurement instrument], Good-Lite. Elgin, IL.

[ref41] Jacobs M. Ellis C. (2021). Estimating the cost and value of functional changes in communication ability following telepractice treatment for aphasia. PLoS One 16:e0257462. doi: 10.1371/journal.pone.0257462, PMID: 34534254 PMC8448307

[ref42] Jaeger T. F. Snider N. E. (2013). Alignment as a consequence of expectation adaptation: syntactic priming is affected by the prime’s prediction error given both prior and recent experience. Cognition 127, 57–83. doi: 10.1016/j.cognition.2012.10.013, PMID: 23354056 PMC7313543

[ref43] Kaplan E. Goodglass H. Weintraub S. (2001). Boston naming test. Philadelphia, PA: Lea and Febiger.

[ref44] Kaschak M. P. (2007). Long-term structural priming affects subsequent patterns of language production. Mem. Cogn. 35, 925–937. doi: 10.3758/BF03193466, PMID: 17910177

[ref45] Kaschak M. P. Kutta T. J. Coyle J. M. (2014). Long and short term cumulative structural priming effects. Lang. Cogn. Neurosci. 29, 728–743. doi: 10.1080/01690965.2011.641387, PMID: 26478892 PMC4608441

[ref46] Kaschak M. P. Kutta T. J. Jones J. (2011). Structural priming as implicit learning: cumulative priming effects and individual differences. Psychon. Bull. Rev. 18, 1133–1139. doi: 10.3758/s13423-011-0157-y, PMID: 21913001 PMC4612612

[ref47] Kaschak M. P. Kutta T. J. Schatschneider C. (2011). Long-term cumulative structural priming persists for (at least) one week. Mem. Cogn. 39, 381–388. doi: 10.3758/s13421-010-0042-3, PMID: 21264596

[ref48] Keen A. D. Lee J. (2022). Structural priming from production to comprehension in aphasia. Aphasiology. doi: 10.1080/02687038.2022.2159314, PMID: 38239274 PMC10794006

[ref49] Kemper S. (1987). Life-span changes in syntactic complexity. J. Gerontol. 42, 323–328. doi: 10.1093/geronj/42.3.323 3571869

[ref50] Kemper S. Herman R. E. Liu C.-J. (2004). Sentence production by young and older adults in controlled contexts. J. Gerontol. Ser. B Psychol. Sci. Soc. Sci. 59, P220–P224. doi: 10.1093/geronb/59.5.P220, PMID: 15358794

[ref51] Kertesz A. (2006). Western aphasia battery–revised (WAB-R), Pro-Ed. Austin, TX.

[ref52] Lee J. Man G. (2017). Language recovery in aphasia following implicit structural priming training: a case study. Aphasiology 31, 1441–1458. doi: 10.1080/02687038.2017.1306638

[ref53] Lee J. Man G. Ferreira V. Gruberg N. (2019). Aligning sentence structures in dialogue: evidence from aphasia. Lang. Cogn. Neurosci. 34, 720–735. doi: 10.1080/23273798.2019.1578890, PMID: 31815155 PMC6897504

[ref54] Lee J. Man G. Keen A. D. Castro N. (2022). Priming sentence production in older adults: evidence for preserved implicit learning. Aphasiology. doi: 10.1080/02687038.2022.2153326 PMC10901520 38425351

[ref55] Lee J. van Boxtel W. S. Weirick J. D. Martin N. Ferreira V. Bauman E.. (2023). Implicit structural priming as a treatment component in aphasia: specifying essential components learning, In Annual meeting of academy of aphasia 2023. 10.1080/02643294.2025.2479485 PMC12140876 40179208

[ref56] Leonard L. Miller C. Grela B. Holland A. Gerber E. Petucci M. (2000). Production operations contribute to the grammatical morpheme limitations of children with specific language impairment. J. Mem. Lang. 43, 362–378. doi: 10.1006/jmla.1999.2689

[ref57] Mahowald K. James A. Futrell R. Gibson E. (2016). A meta-analysis of syntactic priming in language production. J. Mem. Lang. 91, 5–27. doi: 10.1016/j.jml.2016.03.009

[ref58] Man G. Meehan S. Martin N. Branigan H. Lee J. (2019). Effects of verb overlap on structural priming in dialogue: implications for syntactic learning in aphasia. J. Speech Lang. Hear. Res. 62, 1933–1950. doi: 10.1044/2019_JSLHR-L-18-0418, PMID: 31112446 PMC6808374

[ref59] Menenti L. Pickering M. J. Garrod S. C. (2012). Toward a neural basis of interactive alignment in conversation. Front. Hum. Neurosci. 6:24405. doi: 10.3389/fnhum.2012.00185 PMC3384290 22754517

[ref60] Meyer A. M. Getz H. R. Brennan D. M. Hu T. M. Friedman R. B. (2016). Telerehabilitation of anomia in primary progressive aphasia. Aphasiology 30, 483–507. doi: 10.1080/02687038.2015.1081142 27087732 PMC4831866

[ref61] Miller C. Deevy P. (2006). Structural priming in children with and without specific language impairment. Clin. Linguist. Phonetics 20, 387–399. doi: 10.1080/02699200500074339 16728335

[ref62] Øra H. P. Kirmess M. Brady M. C. Sørli H. Becker F. (2020). Technical features, feasibility, and acceptability of augmented telerehabilitation in post-stroke aphasia – experiences from a randomized controlled trial. Front. Neurol. 11:671. doi: 10.3389/fneur.2020.00671 32849176 PMC7411384

[ref63] Palmer K. Monaco A. Kivipelto M. Onder G. Maggi S. Michel J.-P.. (2020). The potential long-term impact of the COVID-19 outbreak on patients with non-communicable diseases in Europe: consequences for healthy ageing. Aging Clin. Exp. Res. 32, 1189–1194. doi: 10.1007/s40520-020-01601-4, PMID: 32458356 PMC7248450

[ref64] Pearson Assessments. (2020). *Administering the Western aphasia battery-revised (WAB-R) via telepractice*. Administering the Western aphasia battery-revised (WAB-R) via telepractice. Available from https://www.pearsonclinical.ca/en/digital-solutions/telepractice/telepractice-and-the-wab-r.html

[ref23] Pickering V. S. Ferreira M. J. (2008). Structural priming: a critical review. Psychol. Bull. 134:427. doi: 10.1037/0033-2909.134.3.427, PMID: 18444704 PMC2657366

[ref65] Pickering M. Garrod S. (2004). Toward a mechanistic psychology of dialogue. Behav. Brain Sci. 27, 169–226. doi: 10.1017/S0140525X04000056 15595235

[ref66] Pickering M. J. Garrod S. (2007). Do people use language production to make predictions during comprehension? Trends Cogn. Sci. 11, 105–110. doi: 10.1016/j.tics.2006.12.002, PMID: 17254833

[ref67] Pitt R. Theodoros D. Hill A. J. Russell T. (2017). The impact of the telerehabilitation group aphasia intervention and networking programme on communication, participation, and quality of life in people with aphasia. Int. J. Speech Lang. Pathol. 21, 513–523. doi: 10.1080/17549507.2018.1488990 30200788

[ref68] Poirier S.-E. Fossard M. Monetta L. (2021). The efficacy of treatments for sentence production deficits in aphasia: a systematic review. Aphasiology 37, 122–142. doi: 10.1080/02687038.2021.1983152

[ref69] Qualtrics. (2020). Qualtrics (version November 2020) [computer software]. Available at: https://www.qualtrics.com

[ref70] Reitter D. Moore J. D. (2014). Alignment and task success in spoken dialogue. J. Mem. Lang. 76, 29–46. doi: 10.1016/j.jml.2014.05.008, PMID: 34307640

[ref71] Rochon E. Laird L. Bose A. Scofield J. (2005). Mapping therapy for sentence production impairments in nonfluent aphasia. Neuropsychol. Rehabil. 15, 1–36. doi: 10.1080/09602010343000327 16353851

[ref72] Saffran E. M. Martin N. (1997). Effects of structural priming on sentence production in aphasics. Lang. Cognit. Processes. 12, 877–882. doi: 10.1080/016909697386772

[ref73] Scheepers C. (2003). Syntactic priming of relative clause attachments: persistence of structural configuration in sentence production. Cognition 89, 179–205. doi: 10.1016/S0010-0277(03)00119-7, PMID: 12963261

[ref74] Schoot L. Hagoort P. Segaert K. (2019). Stronger syntactic alignment in the presence of an interlocutor. Front. Psychol. 10:685. doi: 10.3389/fpsyg.2019.00685 30971995 PMC6445862

[ref75] Smith M. Wheeldon L. (2001). Syntactic priming in spoken sentence production – an online study. Cognition 78, 123–164. doi: 10.1016/S0010-0277(00)00110-4, PMID: 11074248

[ref76] Thompson C.K. (2011). Northwestern assessment of verbs and sentences. Evanston, IL: Northwestern University.

[ref77] Thompson C. K. Shapiro L. (2005). Treating agrammatic aphasia within a linguistic framework: treatment of underlying forms. Aphasiology 19, 1021–1036. doi: 10.1080/02687030544000227 17410280 PMC1847567

[ref78] Thompson C. K. Shapiro L. P. Kiran S. Sobecks J. (2003). The role of syntactic complexity in treatment of sentence deficits in agrammatic aphasia: the complexity account of treatment efficacy (CATE). J. Speech Lang. Hear. Res. 46, 591–607. doi: 10.1044/1092-4388(2003/047), PMID: 14696988 PMC1995234

[ref79] van Boxtel W. S. Cox B. N. Keen A. D. Lee J. (2023). Planning sentence production in aphasia: evidence from structural priming and eye-tracking. Front Lang Sci. 2:1175579. doi: 10.3389/flang.2023.1175579 PMC11601089 39605949

[ref80] Woolf C. Caute A. Haigh Z. Galliers J. Wilson S. Kessie A.. (2016). A comparison of remote therapy, face to face therapy and an attention control intervention for people with aphasia: a quasi-randomised controlled feasibility study. Clin. Rehabil. 30, 359–373. doi: 10.1177/0269215515582074, PMID: 25911523

[ref81] Yan H. Martin R. C. Slevc L. R. (2018). Lexical overlap increases syntactic priming in aphasia independently of short-term memory abilities: evidence against the explicit memory account of the lexical boost. J. Neurolinguistics 48, 76–89. doi: 10.1016/j.jneuroling.2017.12.005

