# Supplementary material for: TelePriming sentence production in aphasia
Source: Front Hum Neurosci. 2023 Nov 9;17:1274620. doi: 10.3389/fnhum.2023.1274620 (PMC10665571; doi:10.3389/fnhum.2023.1274620)
Supplement: Supplementary file 1 [file Table_1.pdf]

Supplemental Material

Study 1:

Table S1. Main Effects Model on the Logit Scale, n=34, o=1,494

	b	p-value	95% CI	
PWA v. YA	-0.84	0.100	-1.84	0.16
OA v. YA	0.96	0.036	0.06	1.85
PWA v. OA	-1.80	0.000	-2.79	-0.80
Verb (same v. different)	0.52	0.005	0.15	0.88
Prime (passive v. active)	3.71	0.000	3.30	4.12
fixed Intercept	-3.93	0.000	-4.67	-3.19
random intercept	1.01		0.53	1.93
random slope Verb	0.32		0.07	1.55

Table S2. Prime by Verb Interaction Model on the Logit Scale, n=34, o=1,494

	b	p-value	95% CI	
PWA v. YA	-0.94	0.063	-1.93	0.05
OA v. YA	0.90	0.046	0.02	1.78
PWA v. OA	-1.84	0.000	-2.83	-0.85
Verb (same v. different)	-1.44	0.001	-2.30	-0.58
Prime (passive v. active)	2.71	0.000	2.24	3.19
Prime X Verb	2.45	0.000	1.55	3.36
fixed Intercept	-3.09	0.000	-3.84	-2.35
random intercept	0.95		0.48	1.88
random slope Verb	0.70		0.24	2.09

Table S3. Group by Prime Interaction Model on the Logit Scale, n=34, o=1,494

	b	p-value	95% CI	
PWA v. YA	-1.62	0.025	-3.04	-0.20
OA v. YA	-0.59	0.310	-1.73	0.55
PWA v. OA	-1.03	0.166	-2.49	0.43
Verb (same v. different)	0.53	0.005	0.16	0.90
Prime (passive v. active)	2.75	0.000	2.19	3.30
PWA v. YA X Prime	0.95	0.109	-0.21	2.12
OA v. YA X Prime	1.94	0.000	1.05	2.82
PWA v. OA X Prime	-0.98	0.120	-2.23	0.26
fixed intercept	-3.14	0.000	-3.92	-2.37
random intercept	0.32		0.07	1.56
random slope Verb	0.99		0.51	1.89

Table S4. Group by Verb by Prime Interaction Model on the Logit Scale, n=34, o=1,494

	b	p-value	95% CI	
PWA v. YA	-1.74	0.033	-3.35	-0.14
OA v. YA	-0.24	0.682	-1.41	0.92
PWA v. OA	-1.50	0.072	-3.14	0.14
Verb (same v. different)	-1.18	0.038	-2.30	-0.06
Prime (passive v. active)	2.02	0.000	1.34	2.70
PWA v. YA X Verb	0.60	0.607	-1.68	2.87
OA v. YA X Verb	-1.63	0.186	-4.04	0.79
PWA v. OA X Verb	2.23	0.137	-0.71	5.16
PWA v. YA X Prime	0.70	0.355	-0.78	2.19
OA v. YA X Prime	1.35	0.009	0.34	2.36
PWA v. OA X Prime	-0.65	0.403	-2.17	0.87
Prime X Verb	1.69	0.004	0.53	2.85
OA v. YA X Verb X Prime	2.54	0.048	0.02	5.06
PWA v. YA X Verb X Prime	0.34	0.779	-2.03	2.71
PWA v. OA X Verb X Prime	-2.20	0.159	-5.26	0.86
fixed Intercept	-2.46	0.000	-3.27	-1.66
random intercept	0.96		0.48	1.90
random slope Verb	0.62		0.18	2.06

Study 2:

Table S5. Main Effects Model OA Group on the Logit Scale, n=24, o=1,132

	b	p-value	95% CI	
Verb (same v. different)	0.78	0.000	0.43	1.13
Prime (passive v. active)	4.12	0.000	3.34	4.90
Study (tele v. in-person)	0.28	0.635	-0.88	1.44
Age	0.02	0.636	-0.05	0.08
fixed Intercept	-4.86	0.047	-9.65	-0.06
random intercept	0.86		0.30	2.44
random slope Prime	1.79		0.69	4.66

Table S6. Main Effects Model PWA Group on the Logit Scale, n=21, o=872

	b	p-value	95% CI	
Verb (same v. different)	0.75	0.001	0.30	1.19
Prime (passive v. active)	2.13	0.000	1.01	3.25
Study (tele v. in-person)	0.68	0.264	-0.51	1.86
Age	0.04	0.282	-0.03	0.10
fixed Intercept	-6.84	0.003	-11.31	-2.36
random intercept	4.67		1.79	12.20
random slope Prime	3.29		1.21	8.92
